# Efficacy and tolerability of the hexanic extract of *Serenoa repens* compared to tamsulosin in moderate-severe LUTS-BPH patients

**DOI:** 10.1038/s41598-021-98586-5

**Published:** 2021-09-29

**Authors:** Antonio Alcaraz, Alfredo Rodríguez-Antolín, Joaquín Carballido-Rodríguez, David Castro-Díaz, José Medina-Polo, Jesús M. Fernández-Gómez, Vincenzo Ficarra, Joan Palou, Javier Ponce de León Roca, Javier C. Angulo, Manuel Esteban-Fuertes, José M. Cózar-Olmo, Noemí Pérez-León, José M. Molero-García, Antonio Fernández-Pro Ledesma, Francisco J. Brenes-Bermúdez, José Manasanch

**Affiliations:** 1 Urology Department, Hospital Clínic, Universitat de Barcelona, IDIBAPS, Barcelona, Spain; 2 Urology Department, Research Group in Men’s Integral Health, Instituto de Investigación i+12, Hospital Universitario 12 de Octubre, Madrid, Spain; 3 Urology Department, Hosp. Univ. Puerta de Hierro Majadahonda, Majadahonda, Madrid, Spain; 4 Urology Department, Hosp. Univ. de Canarias, Tenerife, Spain; 5 Urology Department, Hosp. Univ. Central de Asturias, Oviedo, Spain; 6 Urology Department, University of Messina, Messina, Italy; 7 Urology Department, Fundació Puigvert, Autonoma University of Barcelona, Barcelona, Spain; 8 Urology Department, Hosp. Univ. de Getafe. Getafe, Madrid, Spain; 9 Urology Department, Hosp. Nal. de Parapléjicos, Toledo, Spain; 10 Urology Department, Hosp. Univ. Virgen de Las Nieves, Granada, Spain; 11 Gran Sol Primary Care Center, Badalona, Barcelona, Spain; 12 San Andrés Primary Care Center, Madrid, Spain; 13 Menasalbas Primary Care Center, Toledo, Spain; 14 SEMERGEN Nefro-Urology Working Group, Barcelona, Spain; 15 Pierre Fabre Ibérica S.A., Barcelona, Spain

**Keywords:** Prostate, Benign prostatic hyperplasia, Urological manifestations

## Abstract

In a subset analysis of data from a 6-month, multicenter, non-interventional study, we compared change in symptoms and quality of life (QoL), and treatment tolerability, in men with moderate to severe lower urinary tract symptoms associated with benign prostatic hyperplasia (LUTS/BPH) receiving tamsulosin (TAM, 0.4 mg/day) or the hexanic extract of *Serenoa repens* (HESr, 320 mg/day) as monotherapy. Symptoms and QoL were assessed using the IPSS and BII questionnaires, respectively. Patients in the treatment groups were matched using two statistical approaches (iterative and propensity score matching). Within the iterative matching approach, data was available from a total of 737 patients (353 TAM, 384 HESr). After 6 months, IPSS scores improved by a mean (SD) of 5.0 (4.3) points in the TAM group and 4.5 (4.7) points in the HESr group (*p* = 0.117, not significant). Improvements in QoL were equivalent in the two groups. TAM patients reported significantly more adverse effects than HESr patients (14.7% vs 2.1%; *p* < 0.001), particularly ejaculation dysfunction and orthostatic hypotension. These results show that HESr is a valid treatment option for men with moderate/severe LUTS/BPH; improvements in urinary symptoms and QoL were similar to those observed for tamsulosin, but with considerably fewer adverse effects.

## Introduction

Benign prostatic hyperplasia (BPH) is a non-malignant growth of the prostate tissue and is a frequent cause of lower urinary tract symptoms (LUTS) in men^1,2^. Prevalence of BPH has been estimated at between 50 to 60% for men in their 60s^3^, while prevalence of LUTS associated with BPH (LUTS/BPH) increases from approximately 3% in those aged 45–49 years to over 30% in men aged ≥ 85 years^4^.

The European Association of Urology (EAU) guidelines on the management of LUTS note that symptoms can be divided into 3 types, i.e. storage, voiding and post-micturition symptoms^1^, with voiding symptoms being the most prevalent, but storage symptoms the most bothersome^5^. Several studies have also indicated that LUTS and LUTS/BPH can have a significant impact on patients’ quality of life (QoL)^6,7^ and on the QoL of patients’ partners^5,8^.

Medical treatments most frequently used for LUTS/BPH include alpha-1-adrenergic receptors blockers (AB) and 5-alpha-reductase inhibitors (5ARI), or a combination of both in some patients. Although such treatments have demonstrated their effectiveness in reducing symptoms, they can also negatively affect sexual function, especially ejaculatory function^9^. Other medical treatments also used to treat LUTS/BPH include 5-phosphodiesterase inhibitors, antimuscarinic drugs, beta-3 agonists and phytotherapy. The hexanic extract of *Serenoa repens* (*S. repens*) (HESr) is a phytotherapeutic treatment for LUTS/BPH which has been shown to have anti-inflammatory^10–13^, antiandrogenic^14–17^, and antiproliferative effects^17^.

Improvements in symptoms and QoL in patients treated with the HESr treatment were observed in the non-interventional Quality of Life in Benign Prostatic Hyperplasia, or QUALIPROST study^18^, which assessed the effectiveness and tolerability of commonly used treatments for LUTS/BPH in clinical practice. QUALIPROST provided further evidence that HESr appears to be as effective as AB and 5ARI for the treatment of moderate to severe LUTS/BPH when used as monotherapy or in combination with one of the other treatments over a six-month treatment period^18^ and confirmed the low level of adverse effects associated with HESr treatment, especially in comparison to AB and 5ARI. In the original QUALIPROST publication^18^, it was not possible to analyze in-depth the outcomes in patients treated with tamsulosin (TAM) and compare them with patients receiving the HESr after applying matching techniques.

In this subset analysis of data from the QUALIPROST study, we compared changes in urinary symptoms (overall, voiding and storage) and QoL in men with moderate to severe LUTS/BPH receiving TAM or HESr as monotherapy using two different matching approaches to optimize comparability of the treatment groups. Treatment tolerability was also evaluated and compared.

## Methods

### Patients and study design

Data for this analysis was from the QUALIPROST study (ISRCTN11815680)^18^, a multicenter study to evaluate change in symptoms and QoL in patients with moderate to severe LUTS/BPH (baseline IPSS score > 7 points) managed in a urological setting. The study conformed to the Strengthening the Reporting of Observational Studies in Epidemiology (STROBE) guidelines^19^ and is described in detail in Alcaraz A et al^18^. Briefly, the study used a longitudinal, prospective, non-interventional design in which participants were followed up for a 6-month period. Patients were excluded from QUALIPROST if they had received a medical treatment for BPH in the 6 months prior to inclusion, if they had received any drug treatment with a known effect on BPH symptoms (i.e., diuretics, antihistamines, or tricyclic antidepressants) at any time in the 4 weeks prior to inclusion, if they had other urinary disorders, or if they had previously undergone surgery of the lower urinary tract. As QUALIPROST was a real-world study of patient management, investigators could prescribe any of the commercially available treatments according to their usual practice. A range of treatments were prescribed to manage LUTS/BPH, including monotherapy and combination treatments. The study was conducted according to the guidelines of the Declaration of Helsinki, and approved by the Ethics Committee of the Puerta de Hierro Majadahonda University Hospital in Madrid, Spain. Informed consent was obtained individually from all patients included in the study.

For the present sub-analysis, data was used from patients ≥ 40 years of age with a diagnosis of LUTS/BPH and an IPSS score of > 7 points who had received either TAM (OMNIC, UROLOSIN or generics, at a recommended dose [RD] of 0.4 mg/day) or HESr (PERMIXON; RD: 320 mg/day, 160 mg morning and evening) as monotherapy. PERMIXON contains: free fatty acids, 80.7% (mainly lauric, oleic, myristic and palmitic acids); glycerides, 6.8%; methyl and ethyl esters, 2.5%; unsaponified matter, 2.27%; long-chain esters, 1.36%^20^.

### Study variables

Key endpoints in the QUALIPROST study were change in LUTS evaluated by means of the International Prostate Symptom Score (IPSS) and impact on QoL assessed using the Benign Prostatic Hyperplasia Impact Index (BII). The IPSS includes 8 questions, seven of which assess symptoms of LUTS/BPH, while the eighth assesses QoL associated with the condition. The symptom items assess problems with both storage (urgency, frequency, nocturia) and voiding (incomplete emptying, intermittency, weak stream and straining to void). The overall score on the IPSS ranges from 0 to 35 for the symptom items, with a higher score indicating more severe symptoms, and from 0 to 5 for the QoL item (item 8). An improvement of > 3.1 points on the IPSS questionnaire is considered clinically relevant^21^. Separate sub-scores can also be calculated for the storage and voiding symptoms.

The BII is a self-administered questionnaire consisting of 4 questions measuring the impact of urinary symptoms on physical discomfort, worries about health, symptom bother, and interference with usual activities during the past month^22,23^. Items are answered on a Likert scale, with 4 or 5 response options per item and scores range from 0 (best QoL) to 13 (worst QoL). An improvement of > 0.4 points on the BII questionnaire is considered clinically relevant, as perceived by the patient^21^. Both the BII and the IPSS were self-completed by patients at baseline and at the 6-month follow-up visit.

Sociodemographic data collected at baseline also included age, weight and height, date of onset of urinary symptoms, year of LUTS/BPH diagnosis, results from diagnostic tests when carried out (digital rectal exam, prostate volume, maximum urinary flow, urine analysis, serum analysis, prostate-specific antigen), treatment received (yes/no, alpha-blockers, 5-alpha-reductase inhibitors, phytotherapy, other), and information on co-morbidities and their treatment. Adverse effects potentially associated with treatment were recorded at follow-up.

Treatment compliance was assessed using the validated Spanish version of the Haynes-Sackett questionnaire^24^ which asks about (a) patients’ difficulty taking the medication and (b) the number of tablets they have taken in the previous month. Patients taking 80% or over of the prescribed dose are considered to show good adherence.

### Statistical analysis

#### Optimising comparability: iterative and propensity score matching

To optimise comparability between the TAM and HESr groups, we used two different approaches to select the sample to be included from each group. Initially, an iterative matching procedure was used to ensure comparable group mean scores for baseline IPSS (total score, and voiding and storage subscores), QoL (IPSS item 8) and BII scores, maximum urinary flow (Qmax), prostate-specific antigen (PSA), and prostate volume. The iterative matching procedure is intended to ensure that two or more study groups are comparable in terms of a given set of variables. This approach selects individuals consecutively from one or more of the groups in such a way that when the means of specific variables are compared between groups, the result is not significant (i.e. when performing a t-test, the resulting *p* value is not significant, using a type I error of 10%). Thus, the two groups are matched in terms of means of the variables used to guide the iterative matching (IPSS, BII, etc.). Unlike propensity score matching (another standard technique to select individuals and make groups comparable), with iterative matching the groups may contain different numbers of patients, which helps to guarantee the largest possible sample size. In line with this methodology, therefore, patients were removed one by one from one of the two groups and the two groups continually compared until there were no statistically significant differences (*p* > 0.1) between them on any of the selected baseline characteristics listed above.

As further confirmation of the study results, we carried out a propensity score matching procedure, whereby a propensity score was calculated for each patient for inclusion in either treatment group, again based on baseline IPSS (total score, and voiding and storage subscores), QoL (IPSS item 8) and BII scores, Qmax, PSA, and prostate volume. Each patient in the TAM group was then paired with a patient from the HESr group with a similar propensity score (within a pre-established range of ± 0.2 SD), giving groups of equal size for comparison.

The iterative matching procedure was performed using t-tests to compare treatment groups in each elimination round and Student’s t-test was used to determine the success of the matching procedure, by testing for post-matching baseline between-group differences on the IPSS and BII total scores, the IPSS voiding and storage sub-scores, and IPSS item 7 (nocturia). Matching was done at both the level of the overall sample and for sub-groups of patients defined by IPSS severity.

#### Assessing change over time

Change over time within the different treatment groups was assessed using paired t-tests and between-group differences in the size of change on the IPSS and BII was assessed by t-tests for independent samples. Changes on items 1–7 on the IPSS, which assess symptom severity, were analyzed separately from item 8, which assesses QoL. Outcomes on the IPSS storage and voiding sub-scores were also analyzed and compared between groups.

#### Responder analysis

A responder analysis of patients was performed using results on IPSS items 1–7 and the results compared between groups using the chi-squared test. Responders were defined as patients who improved by 3.1 points or more on the IPSS questionnaire, a change which is considered clinically relevant^21^. The same analysis was repeated for patients showing an improvement of ≥ 25% in their IPSS score and for patients showing a worsening in symptoms (increase of 4 points or more on the IPSS overall score).

#### Adverse effects

Adverse effects were analyzed in terms of frequencies and proportions and compared between groups using the chi-square or exact Fisher test as appropiate.

#### Sub-group analysis

All analyses were performed for the sample as a whole and for two sub-groups defined by baseline severity of urinary symptoms, i.e. a moderate group with a baseline IPSS score of 8–19 and a more severe group with a baseline IPSS score of ≥ 20 points.

Patients with any missing data on the IPSS and BII at any visit were excluded from the analysis as were any patients who were lost to follow-up or that stopped or changed treatment. In all comparisons, results were considered statistically significant at *p* < 0.05. Statistical analyzes were carried out using R 3.5.2 statistical software^25^.

## Results

### Results based on iterative matching: available data and study flow chart

**Figure 1:**
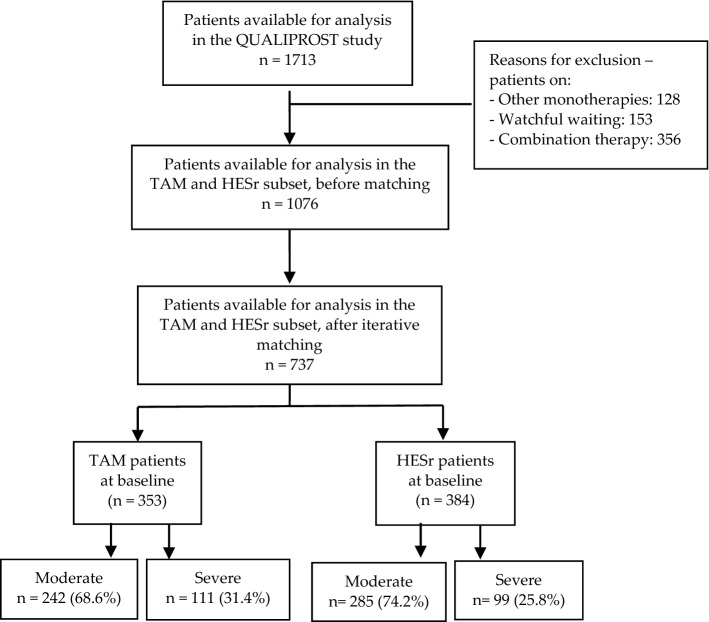
Study flow-chart: iterative matching based on IPSS (total, voiding and storage sub-scores, and item 8) and BII scores, maximum urinary flow rate (Qmax), prostate-specific antigen (PSA), and prostate volume at baseline. TAM: tamsulosin; HESr: hexanic extract of *Serenoa repens*.

After the iterative matching procedure, data was available from 737 patients (353 for TAM and 384 for HESr). The number and proportion of patients reporting moderate or severe IPSS at baseline in the TAM and the HESr groups are shown in Fig. 1.

### Sociodemographic and clinical characteristics of the sample after iterative matching

Table 1 shows the sociodemographic and clinical characteristics for the treatment groups at baseline for the matched samples. At baseline, mean IPSS (SD) was 17.4 (4.8) in the tamsulosin group and 16.8 (5.2) in the HESr group (*p* = 0.105) There were no statistically significant differences between the two groups on any of the clinical parameters analyzed, either overall or when analyzed according to baseline IPSS intensity (moderate or severe). Similarly, there were no statistically significant differences between groups in the frequency of recorded concomitant diseases (Supplementary Table S1). Mean total baseline IPSS was slightly higher in older patients, with a mean (SD) IPSS score of 16.3 (4.7) points in patients ≤ 65 years compared to a mean of 17.6 (5.1) points in those aged > 65 years.

**Table 1:** Patient baseline characteristics by treatment group (iterative matching sample).

	TAM		HESr		*p* value
	n	Mean (SD)	n	Mean (SD)	
Age, mean (SD) years	318	64.6 (8.4)	335	63.5 (9.1)	0.112
BMI (kg/m^2^), mean (SD)	311	26.8 (2.9)	332	26.7 (2.9)	0.487
**IPSS, mean (SD)**	353	17.4 (4.8)	384	16.8 (5.2)	0.105
IPSS voiding subscore	353	10.0 (3.3)	384	9.6 (3.4)	0.109
IPSS storage subscore	353	7.4 (2.2)	384	7.2 (2.4)	0.232
Nocturia	353	2.4 (1.0)	384	2.4 (1.0)	0.539
BII, mean (SD)	353	7.2 (2.3)	384	6.9 (2.3)	0.107
IPSS 8 (QoL)	353	3.7 (1.1)	384	3.6 (1.1)	0.163
Time since diagnosis (years)	317	1.0 (2.3)	334	1.3 (2.9)	0.167
Qmax (ml/s)	159	11.8 (3.4)	158	12.4 (3.6)	0.111
Prostate volume (cm^3^)	301	51.7 (19.5)	324	49.3 (17.0)	0.109
PSA (ng/ml)	325	2.5 (1.3)	351	2.4 (1.2)	0.375

TAM: tamsulosin; HESr*:* hexanic extract of *Serenoa repens.* BMI: body mass index; IPSS: International Prostate Symptom Score; BII: Benign Prostatic Hyperplasia Impact Index; QoL: quality of life; Qmax: maximum urinary flow rate; PSA: prostate-specific antigen.

### Change in symptoms, QoL and clinical parameters after 6 months (iterative matching sample)

Table 2 shows the change in symptoms, QoL and clinical parameters for the TAM and HESr groups after 6 months of treatment. The mean (SD) change in IPSS score was 5.0 (4.3) points for those treated with tamsulosin and 4.5 (4.7) for patients receiving HESr (*p* = 0.117); mean (SD) improvement on the BII improvement was 2.3 (2.4) and 2.2 (2.5) points, respectively (*p* = 0.417). Analysis of IPSS scores showed similar improvements in voiding and storage symptoms for both treatments. There were no statistically significant differences between the groups in any of these outcomes.

**Table 2:** Improvements from baseline to 6-month follow-up in symptoms and quality of life by treatment group (iterative matching sample).

	TAM		HESr		*p* value
	n	Mean (SD)	n	Mean (SD)	
**IPSS total**	335	5.0 (4.3)	369	4.5 (4.7)	0.117
IPSS voiding sub-score	335	2.9 (2.8)	369	2.5 (3.1)	0.051
IPSS storage sub-score	335	2.1 (2.1)	369	2.0 (2.3)	0.527
Nocturia	335	0.6 (0.9)	369	0.6 (1.0)	0.539
BII total	335	2.3 (2.4)	369	2.2 (2.5)	0.417
IPSS 8 (QoL)	335	1.3 (1.2)	369	1.1 (1.2)	0.129

TAM: tamsulosin; HESr*:* hexanic extract of *Serenoa repens;* IPSS: International Prostate Symptom Score; BII: Benign Prostatic Hyperplasia Impact Index; QoL: quality of life.

Data on change in Qmax, prostate volume and PSA for patients who underwent these tests at both baseline and the 6-month follow-up visit in the two study groups is provided in Table 3. Given that this was an observational study in which clinicians applied their usual criteria for requesting Qmax and PSA analysis, patient numbers with available data for those tests were considerably lower than those with available data on the IPSS and BII, both at baseline and follow-up. Based on the available results, the mean improvement in Qmax ranged from 3.1 to 3.2 ml/s and the mean change in prostate volume was about − 2.6 cm^3^ for both arms. Similarly, the change in PSA values between the study groups available at follow-up was 0.2 and 0.1 ng/ml for the TAM and the HESr groups, respectively. No statistically significant differences were found in these outcomes.

**Table 3:** Change from baseline to 6-month follow-up in PSA, Qmax and prostate volume for the study groups (iterative matching sample).

	TAM		HESr		p value
	n*	Mean (SD)	n*	Mean (SD)	
PSA total (ng/ml)	116	− 0.2 (1.3)	128	− 0.1 (0.8)	0.771
Qmax (ml/sec)	62	3.2 (3.4)	79	3.1 (3.6)	0.849
Prostate volume (cm^3^)	61	− 2.6 (9.3)	89	− 2.7 (11.6)	0.961

TAM: tamsulosin; HESr*:* hexanic extract of *Serenoa repens;* Qmax: maximum urinary flow rate; PSA: prostate-specific antigen.

*Number of patients vary according to the test and the personal clinical practice of the investigators.

### Change in IPSS score based on baseline symptom severity (iterative matching sample)

**Figure 2:**
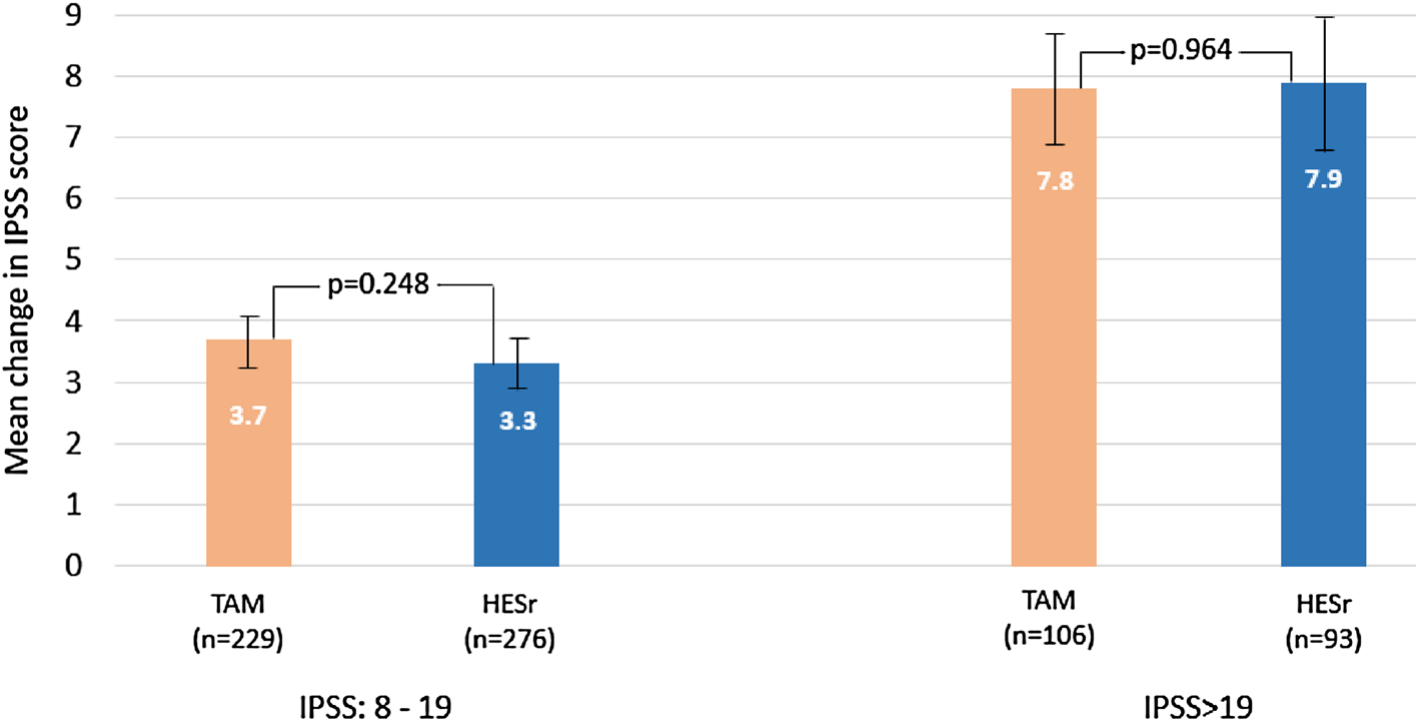
Mean change (95% CI) in IPSS total score from baseline to 6 months for the treatment groups based on baseline symptom severity (iterative matching sample). TAM: tamsulosin; HESr: hexanic extract of *Serenoa repens;* IPSS: International Prostate Symptom Score.

When change in IPSS scores was analyzed by baseline symptom severity (Fig. 2 and Table 4), patients in both the moderate and severe baseline symptom groups showed improvement, with no statistically significant differences between the TAM and HESr groups. Gains were larger in patients with more severe baseline symptoms (IPSS mean improvement of 7.8 and 7.9 points for TAM and HESr, respectively, in the more severe group, compared to 3.7 and 3.3 points, respectively, in patients with moderate baseline symptoms). Baseline and follow-up IPSS scores according to sub-groups defined by baseline severity (IPSS moderate or severe) are provided in Supplementary Figure S2.

**Table 4:** Improvements from baseline to 6-month follow-up in symptoms, quality of life, and clinical parameters by treatment group, in patients with severe (IPSS > 19) baseline symptoms (iterative matching sample).

	TAM		HESr		*p* value
	n	Mean (SD)	n	Mean (SD)	
**IPSS total**	106	7.8 (4.9)	93	7.9 (5.3)	0.964
IPSS voiding sub-score	106	4.7 (3.1)	93	4.7 (3.2)	0.977
IPSS storage sub-score	106	3.1 (2.4)	93	3.2 (2.5)	0.955
Nocturia	106	1.0 (1.0)	93	1.1 (1.1)	0.673
BII total	106	2.8 (2.7)	93	2.8 (3.2)	0.968
IPSS 8 (QoL)	106	1.6 (1.2)	93	1.3 (1.3)	0.184

HESr*:* hexanic extract of *Serenoa repens;* TAM: tamsulosin; IPSS: International Prostate Symptom Score; BII: Benign Prostatic Hyperplasia Impact Index; QoL: quality of life.

A similar pattern was seen when results were analyzed by symptom type (storage or voiding). In patients with moderate baseline symptoms, the IPSS storage symptom sub-score improved by 1.6 points (*p* = 0.947) in both treatment groups, compared to an improvement of 3.1 and 3.2 points (*p* = 0.955) in the TAM and HESr groups, respectively, in patients with more severe baseline symptoms. On the voiding sub-score, patients with moderate baseline symptoms treated with TAM and HESr showed improvements of 2.1 and 1.8 points (*p* = 0.098), respectively, while those with more severe baseline symptoms showed an improvement of 4.7 points (*p* = 0.977) in both treatment groups. There were no statistically significant differences between the groups on these analyses. When change in IPSS score was analysed across different age groups according to treatment type, no statistically significant differences were observed between tamsulosin and the HESr in terms of efficacy in any of the age groups (50–60; 61–70; 71–80 years).

**Figure 3:**
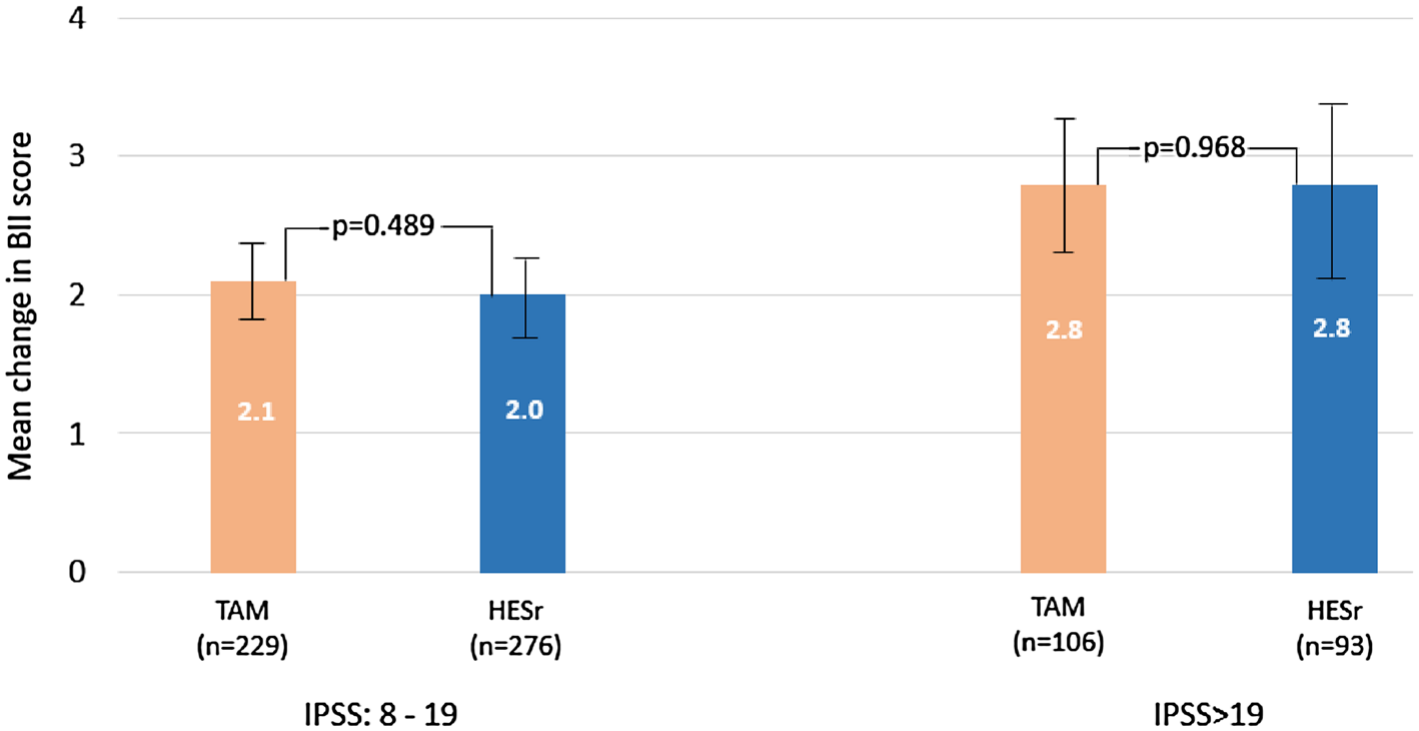
Mean improvement (95% CI) in mean BII total score by baseline symptom severity and treatment group, baseline to 6 months (iterative matching sample). TAM: tamsulosin; HESr: hexanic extract of *Serenoa repens;* BII: Benign Prostatic Hyperplasia Impact Index; IPSS: International Prostate Symptom Score.

Based on the iterative matching samples, QoL improved to a similar degree in both treatment groups, both when patients were assessed overall (mean [SD] improvement in BII score of 2.3 [2.4] points for TAM and 2.2 [2.5] points for HESr) and when analyzed by baseline symptom severity. As shown in Fig. 3, patients with more severe baseline symptoms showed greater improvement. Supplementary Figure S3 shows the mean baseline and follow-up BII scores according to sub-groups defined by baseline symptom severity (IPSS 8—19; IPSS > 19).

A similar pattern was observed when item 8 of the IPSS was used to assess QoL, with a mean [SD] improvement of 1.1 [1.2] and 1.0 [1.2] points in the TAM and HESr groups, respectively, in patients with moderate baseline symptoms (*p* = 0.535), and an improvement of 1.6 [1.2] and 1.3 [1.3] points, respectively, in the more severe baseline patients (*p* = 0.184). A statistically significant (*p* < 0.001) correlation of 0.570 and 0.569 for the TAM and HESr arms, respectively, was observed between the BII total score and question 8 of the IPSS.

Results of the IPSS responder analysis showed that 240 (71.6%) patients in the TAM group improved by at least 3 points on the IPSS compared to 242 (65.6%) of patients in the HESr group (*p* = 0.100).

### Treatment compliance

With respect to treatment compliance, 31 (9.4%) of TAM patients reported difficulty taking the medication compared to 35 HESr patients (9.7%), with no statistically significant difference between the two groups (*p* = 0.976). Among the patients that did report any difficulty taking the medication, over 93.5% and 91.4% for the TAM and HESr groups, respectively, reported good treatment adherence, with no statistically significant differences between them (*p* = 1.000).

### Adverse effects

Table 5 shows the incidence of adverse effects (AE) overall and for the two treatment groups. 52 (14.7%) patients reported at least one AE in the TAM group compared to 8 (2.1%) patients in the HESr group (*p* < 0.001). The most frequent AE was anejaculation (reported by 8.5% of patients receiving TAM, compared to 0 patients on HESr; *p* < 0.001). This was followed by reduced ejaculatory volume (5.1% of patients on TAM and 0.5% of HESr patients; *p* < 0.001), and orthostatic hypotension (reported by 2% of TAM patients and no HESr patients; *p* = 0.006).

**Table 5:** Reported adverse effects for the study sample overall and by treatment group.

	Overall	TAM	HESr	*p* value
	n = 737	n = 353	n = 384	
Any adverse effect	60 (8.1%)	52 (14.7%)	8 (2.1%)	< 0.001*
Anejaculation	30 (4.1%)	30 (8.5%)	0	< 0.001*
Reduced ejaculatory volume	20 (2.7%)	18 (5.1%)	2 (0.5%)	< 0.001*
Orthostatic hypotension	7 (1.0%)	7 (2.0%)	0	0.006*
Dizziness	7 (1.0%)	4 (1.1%)	3 (0.8%)	0.715
Reduced libido	5 (0.7%)	4 (1.1%)	1 (0.3%)	0.199

Only AE with an incidence of ≥ 1% in any of the groups are reported (iterative matching sample).

TAM: tamsulosin; HESr: hexanic extract of *Serenoa repens.*

*Statistically significant.

### Results based on propensity matched samples

After propensity matching, data was available for a total of 128 patients each for TAM and HESr. Of those, 93 patients had moderate symptoms and 35 had severe symptoms at baseline in the TAM group, compared to 100 and 28 patients, respectively, in the HESr group (Supplementary Figure S1). Mean (SD) baseline IPSS was 16.7 (4.5) and 16.3 (4.9) points in the TAM and HESr groups, respectively, with no statistically significant differences between the groups at baseline (*p* = 0.579). Mean (SD) baseline BII was 7.2 (2.2) and 7.0 (2.4) points, respectively, for TAM and HESr, with no statistically significant differences between the groups at baseline (*p* = 0.499). There were no statistically significant differences between the two treatment groups on any other baseline characteristics (Supplementary Table S2).

As in the analysis based on the iterative matching procedure, overall IPSS and BII scores showed a similar level of improvement in symptoms and QoL, with no statistically significant differences between the groups in terms of the size of the improvement. Mean (SD) change on the IPSS was 5.1 (4.6) points in the TAM group and 4.9 (4.6) in HESr patients (*p* = 0.718 for the difference in the size of the change between the two groups). On the BII, mean change was 2.6 (2.4) points for TAM patients and 2.4 (2.5) points for HESr patients (*p* = 0.494 for the difference in the size of the change between the two groups). There were no statistically significant differences between the treatment groups on any of the other outcomes analysed (Supplementary Table S3).

In patients with more severe baseline symptoms (IPSS > 19), similar levels of change were observed in both treatment groups on all endpoints assessed, with no statistically significant differences between groups (Supplementary Table S4). For example, mean (SD) improvement in IPSS total score was 9.1 (5.3) in the TAM group and 9.0 (5.4) in the HESr group (*p* = 0.929 for the difference in the size of the change between the two groups), while mean (SD) change on the BII was 3.3 (2.8) in the TAM group and 3.6 (2.8) in the HESr group (*p* = 0.593 for the difference in the size of the change between the two groups). Likewise, the overall incidence of AEs was higher in the TAM group (21 patients, 16.4%) than in the HESr group (3 patients, 2.3%) and the difference was statistically significant at *p* < 0.001 (Supplementary Table S5). Anejaculation and reduced ejaculatory volume were the two individual AEs showing statistically significant between-group differences in incidence favoring the HESr (*p* = 0.002 and *p* = 0.014, respectively).

## Discussion

As far as we know, this is the first prospective, non-interventional, 6-month follow-up study to compare TAM and HESr in current clinical practice which includes an specific assessment of change in QoL using the BII questionnaire. Investigating treatment effectiveness in conditions of usual clinical practice is important because it provides complementary evidence to that obtained in randomized controlled trials (RCTs)^26^. RCTs reported by Debruyne et al^27^, which showed similar efficacy for the HESr (PERMIXON) and tamsulosin in providing relief from symptoms, or that reported by Latil et al^28^, which showed that HESr reduced inflammatory activity in the prostate to a greater extent than tamsulosin, are clearly important but the results may not always translate to clinical practice. The QUALIPROST Study was performed in the conditions and with the type of patients regularly found in clinical practice. On the other hand, as real-world studies like QUALIPROST are not performed under such controlled conditions as RCTs, between-group matching techniques are useful in reducing possible bias when comparing results across treatments.

The present subset analysis is also relevant because it analyzes one specific *Serenoa repens* extract, in line with the 2020 European Association of Urology guidelines on the management of LUTS^1^ which recommended that results from different clinical trials should be compared strictly according to the same validated extraction technique and/or content of active compounds^29^. The recommendation is based on the fact that the pharmacokinetic properties of different preparations can vary significantly so extracts of the same plant produced by different companies may not have the same biological or clinical effects^1^. Indeed, Habib and colleagues have shown that different *Serenoa repens* extracts can vary considerably in composition^20^. Potency has also been shown to vary across commercially available plant extracts, with some being no more effective than placebo^30^ The HESr is the only *S. repens* extract which the European Medicines Agency (EMA) considers as having sufficient evidence to support its use as treatment for LUTS/BPH^31^. In support of the EMA’s position, two recently published, exhaustive systematic reviews showed that the hexanic extract of *S. repens* (HESr) reduces nocturia and improves Qmax compared with placebo, and that, in terms of efficacy, it is similar to tamsulosin and short-term 5ARI in relieving LUTS^32,33^ and the recently published 2021 EAU guidelines on the management of LUTS recommends offering hexane extracted *Serenoa repens* to men with LUTS who want to avoid any potential adverse events especially related to sexual function^34^.

The present subset analysis of matched data from the QUALIPROST Study provides evidence that men with moderate/severe LUTS/BPH treated for 6 months with HESr show similar levels of improvement in urinary symptoms and QoL as those treated with tamsulosin, but that the HESr was associated with fewer adverse effects. These results further demonstrate the HESr’s efficacy and support findings from RCTs that have compared HESr and TAM. In an RCT comparing TAM and the HESr in men with a baseline IPSS ≥ 10, Debruyne et al^27^ reported a 4.4-point improvement in total IPSS in both groups after 12 months of treatment, which is very similar to our findings of a mean improvement in IPSS of 4.5 points in the HESr group and 5 points in the TAM group after 6 months of treatment. In the CombAT trial, Barkin et al^35^ also observed improvements of 4.7 points in total IPSS after 9 months of treatment with tamsulosin.

In further analysis focusing on patients with severe baseline urinary symptoms, Debruyne et al^36^ found that total IPSS improved by 7.8 points with PERMIXON after 12 months of treatment compared to 5.8 points with tamsulosin. These results align with our finding of a mean improvement of 7.9 points for patients in the HESr group, though we observed a mean improvement of 8.8 points in the severe patients treated with TAM. All of the improvements seen on the IPSS in our study, in both the overall sample and the more severe patients, exceed the minimal relevant difference of 3.1 points which has been established for the IPSS total score^21^. Similarly, the mean decrease of ≥ 2.2 points reported on the BII questionnaire in all the groups in this analysis represents a marked clinical improvement in QoL experienced by the patients, according to the Barry et al. threshold^21^.

The improvements in QoL presented here are also similar to those observed in earlier studies. In the CombAT trial, after 6 months of treatment with tamsulosin (mean baseline BII score: 5.3 points) the BII showed an adjusted mean improvement of 1.5 points^35^ compared to a mean improvement of 2.3 points for TAM (mean baseline BII score: 7.2 points) and of 2.2 points for the HESr (mean baseline BII score: 6.9 points) in our study. The adjusted mean improvement on IPSS Q8 in the CombAT study was 0.9 points in the tamsulosin arm (mean baseline IPSS for Q8: 3.6 points), compared to 1.3 in our study for tamsulosin (mean baseline BII score: 3.7 points) and 1.1 points for the HESr (mean baseline BII score: 3.6 points).

The difference between our study and many of the results reported previously is that we observed these changes in conditions of usual clinical practice, for example in patients with concomitant diseases, rather than in the carefully controlled conditions of an RCT. Those controlled conditions, and the fact that RCTs are often carried out in a narrower range of patients than is seen in usual practice, can limit the external validity of their results. Real world studies, on the other hand, are useful because they provide complementary evidence on treatments in conditions where factors such as poor adherence, or the presence of concomitant conditions can impact effectiveness^26^. This is of relevance in the present analysis, as tamsulosin is the most commonly prescribed alpha-blocker in recent years^37^.

As observed in several previous studies, one advantage of the HESr is the low rate of associated adverse effects. Indeed, 2.1% of patients receiving HESr in this study reported any AE compared to 14.7% in the TAM group. Adverse effects in the TAM group primarily affected sexual function, which may be an area of particular concern to the men who take the medication. In terms of treatment safety, alpha-blockers such as tamsulosin or silodosin are considered in the FORTA (LUTS-Fit fOr The Aged) 2014 classification^38^ to be of questionable use (FORTA C) in patients aged 65 or over, and it is suggested that other alpha-blockers, including alfuzosin, doxazosin, or terazosin, should be avoided in that age group (FORTA D). The HESr and other phytotherapeutic drugs were not evaluated in the 2014 FORTA classification, as only the most widely used drugs for LUTS-BPH were included in this classification. Furthermore, in a recent cohort study among patients aged ≥ 65 years and diagnosed with BPH, the tamsulosin cohort (n = 253,136 patients) was associated with a significantly higher risk of dementia when compared with no-BPH-medication cohort and with the other alternative-BPH-medication cohorts evaluated^39^.

As treatment strategy should aim to maximise clinical benefit while limiting side effects, then treatment for LUTS/BPH should be tailored to the individual patient’s symptomatology, comorbidities, and preferences, and take into account treatment tolerability. Discussion of the risk of adverse effects with patients prior to prescription treatments for LUTS/BPH is clearly important. Tamsulosin treatment has been associated with ejaculatory dysfunction (EjD)^9,40^, with a higher rate of EjD than the nonselective alpha-1-adrenergic receptor antagonists^41^ and an incidence of EjD up to 26% depending on dose and treatment duration^42^. It has also been reported that almost 90% of healthy volunteers taking 0.8 mg of tamsulosin showed decreased ejaculate volume and 35.4% had complete anejaculation^43^, which is particularly important if preservation of fertility is desired.

Strengths of the present study include the matching approach, which, as indicated by the lack of any statistically significant differences between groups at baseline, ensured a high level of comparability between the treatment arms. The inclusion of the propensity score analysis helps to further reduce the possibility of bias in the study and provides additional support for the robustness of the results.

The present study has some limitations. Data were obtained using a non-interventional design without randomization or blinding. Patients were therefore allocated to a specific management approach based on clinician judgement, which could lead to a selection bias. Nevertheless, as noted, considerable steps were taken in analysis to reduce the risk of bias. The 6-month follow-up period could also be considered a study limitation. Nevertheless, this time period is likely to be sufficient to observe the effects of TAM, as it has been reported to have a rapid onset of action, with maximum effect observed within the first 3 months of treatment^27,44^. In the case of the HESr, the results observed appear to be due to a combination of its proven anti-inflammatory, 5-alpha-reductase inhibition and antiproliferative mechanisms of action, although more time might be required to observe the full effect of the 5-alpha-reductase inhibition mechanism. In fact, a 2-year clinical study showed that results after 24 months of treatment with the HESr were 10.5% better than those observed at 6-months^45^. Therefore, although clinically relevant improvements in IPSS were seen in the HESr group after 6 months in the current study, a longer follow-up could potentially achieve slightly better results. It is also of note that some in vitro studies in rodents have shown *Serenoa repens* to have a smooth muscle relaxant effect in the prostate^46^ and bladder^47^, which might also contribute to its efficacy. Finally, it would be of interest to investigate whether the extension of the symptoms and QoL improvements over a longer period could be associated with a reduction in the risk of progression and whether the higher rate of AEs in the TAM group could affect long-term quality of life, cost of treatment, and treatment adherence.

In conclusion, a range of treatment options are available for patients with moderate-severe LUTS/BPH, with differing characteristics in terms of the degree of symptom relief achieved, tolerability, and impact on QoL. The availability of different options means that treatment of LUTS/BPH can be customised to the individual patient’s symptomatology, comorbidities, and preferences. Beginning treatment with the least aggressive option in terms of tolerability is a reasonable approach to symptom management, as long as that option aligns with patient preferences and has demonstrated effectiveness. In the present analysis, the hexanic extract of *S. repens* appears to have similar efficacy to tamsulosin but with better tolerability which likely makes it a good first-line treatment choice for many patients.

## Supplementary Information

Supplementary Information.

## Data Availability

The data presented in this study are available on reasonable request from the corresponding author.
