# Supplementary material for: Efficacy and tolerability of the hexanic extract of Serenoa repens compared to tamsulosin in moderate-severe LUTS-BPH patients
Source: Sci Rep. 2021 Sep 29;11:19401. doi: 10.1038/s41598-021-98586-5 (PMC8481292; doi:10.1038/s41598-021-98586-5)
Supplement: Supplementary file 1 — Supplementary Information. [file 41598_2021_98586_MOESM1_ESM.pdf]

Supplementary Information

Table S1. Concomitant diseases at baseline by treatment group (iterative matching sample), n (%).

	TAM (n = 353)	HESr (n = 384)	p value
Patients with any concomitant illness, n (%)	131 (37.1)	120 (31.2)	0.110
High blood pressure	68 (19.3)	60 (15.6)	0.228
Dyslipidemia	55 (15.6)	50 (13.0)	0.375
Diabetes mellitus	38 (10.8)	32 (8.3)	0.318
Other	8 (2.3)	8 (2.1)	0.604

TAM: tamsulosin; HESr: hexanic extract of *Serenoa repens*

Table S2. Patient baseline characteristics by treatment group (propensity score matching sample).

	TAM		HESr		p value
	**n**	**mean (SD)**	**n**	**mean (SD)**	
Age, mean (SD) years	115	63.1 (8.0)	114	62.7 (8.2)	0.713
BMI (Kg/m^2), mean (SD)	113	26.9 (2.8)	114	26.4 (2.6)	0.122
IPSS, mean (SD)	128	16.7 (4.5)	128	16.3 (4.9)	0.579
IPSS voiding sub-score	128	9.6 (3.2)	128	9.3 (3.1)	0.448
IPSS storage sub-score	128	7.1 (2.0)	128	7.1 (2.6)	0.914
Nocturia	128	2.4 (0.9)	128	2.3 (1.0)	0.520
BII, mean (SD)	128	7.2 (2.2)	128	7.0 (2.4)	0.499
IPSS item 8 (QoL)	128	3.5 (1.1)	128	3.4 (1.1)	0.465
Time since diagnosis (years)	115	0.8 (1.6)	114	1.3 (3.0)	0.094
Qmax (ml/s)	128	12.0 (3.4)	128	12.5 (3.5)	0.300
Prostate volume (cm^3)	128	49.5 (16.8)	128	47.7 (15.6)	0.352
PSA (ng/ml)	128	2.3 (1.2)	128	2.3 (1.2)	0.932

TAM: tamsulosin; HESr: hexanic extract of *Serenoa repens.* BMI: body mass index; IPSS: International Prostate Symptom Score; BII: Benign Prostatic Hyperplasia Impact Index; QoL: quality of life; Qmax: maximum urinary flow rate; PSA: prostate-specific antigen.

Table S3. Improvements in symptoms, quality of life, and clinical parameters from baseline to 6-month follow-up by treatment group (propensity score matching sample).

	TAM		HESr		p value
	**n**	**Mean (SD)**	**n**	**Mean (SD)**	
IPSS total	124	5.1 (4.6)	125	4.9 (4.6)	0.718
IPSS voiding sub-score	124	3.0 (3.0)	125	2.7 (2.9)	0.400
IPSS storage sub-score	124	2.1 (2.1)	125	2.2 (2.2)	0.691
Nocturia	124	0.7 (0.9)	125	0.7 (1.0)	0.964
BII total	124	2.6 (2.4)	125	2.4 (2.5)	0.494
IPSS item 8	124	1.3 (1.2)	125	1.2 (1.2)	0.559
Qmax (ml/sec)	51	3.2 (3.6)	65	3.0 (4.2)	0.805
Prostate volume (cm^3)	20	-1.9 (3.4)	36	-2.3 (10.6)	0.824
PSA (ng/ml)	42	-0.3 (0.7)	35	0.0 (0.9)	0.127

TAM: tamsulosin; HESr: hexanic extract of *Serenoa repens;* IPSS: International Prostate Symptom Score; BII: Benign Prostatic Hyperplasia Impact Index; Qmax: maximum urinary flow rate; PSA: prostate-specific antigen.

Table S4. Improvements from baseline to 6-month follow-up in symptoms and quality of life scores by treatment arm, in patients with severe (IPSS > 19) baseline symptoms (propensity score matching sample).

	TAM		HESr		p value
	**n**	**mean (SD)**	**n**	**mean (SD)**	
IPSS total	35	9.1 (5.3)	28	9.0 (5.4)	0.929
IPSS voiding sub-score	35	5.6 (3.2)	28	5.3 (3.3)	0.731
IPSS storage sub-score	35	3.5 (2.6)	28	3.7 (2.5)	0.799
Nocturia	35	1.1 (1.1)	28	1.0 (1.2)	0.633
BII total	35	3.3 (2.8)	28	3.6 (2.8)	0.593
IPSS item 8	35	1.8 (1.2)	28	1.5 (1.3)	0.306
Qmax (ml/sec)	16	3.5 (3.1)	18	5.5 (3.9)	0.103

TAM: tamsulosin; HESr: hexanic extract of *Serenoa repens;* IPSS: International Prostate Symptom Score; BII: Benign Prostatic Hyperplasia Impact Index; Qmax: maximum urinary flow rate.

Table S5. Reported adverse effects for the study sample overall and by treatment group (propensity score matching sample).

	**Overall**	**TAM**	**HESr**	**p value**
	n = 256	n = 128	n = 128	
Any adverse effect	24 (9.4%)	21 (16.4%)	3 (2.3%)	<0.001*
Anejaculation	11 (4.3%)	11 (8.6%)	0	0.002*
Reduced ejaculatory volume	11 (3.4%)	10 (7.8%)	1 (0.8%)	0.014*
Erectile dysfunction	2 (0.8%)	1 (0.8%)	1 (0.8%)	1.000
Orthostatic hypotension	3 (1.2%)	3 (2.3%)	0	0.247
Reduced libido	4 (1.6%)	3 (2.3%)	1 (0.8%)	0.622
Dizziness	1 (0.4%)	1 (0.8%)	0	1.000

TAM: tamsulosin; HESr: hexanic extract of *Serenoa repens*
*Statistically significant

Supplementary figure

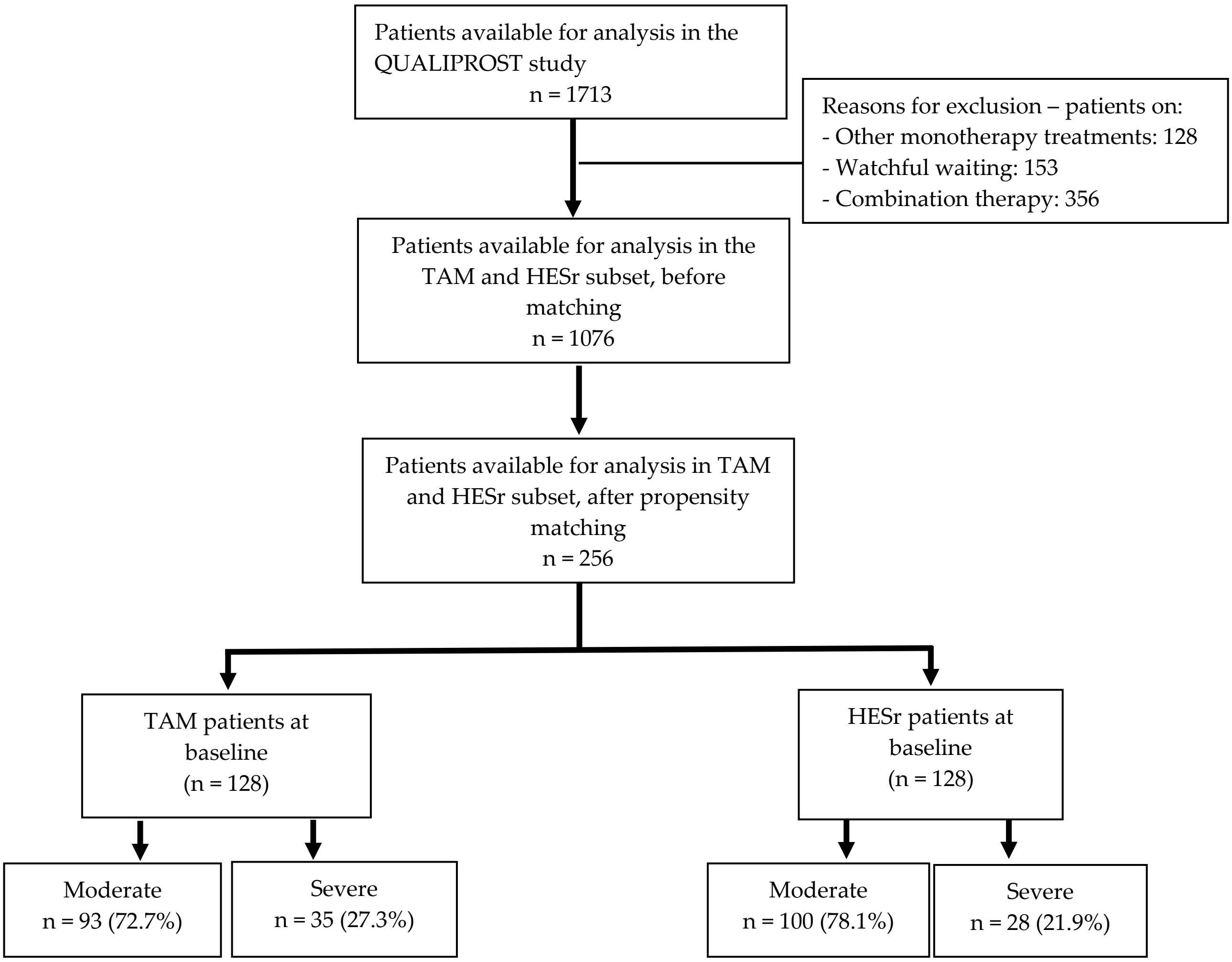

Figure S1. Study flow-chart: propensity matching based on IPSS (total, voiding and storage sub-scores, and item 8) and BII scores, maximum urinary flow (Qmax), prostate-specific antigen (PSA), and prostate volume at baseline.
TAM: tamsulosin; HESr: hexanic extract of *Serenoa repens*

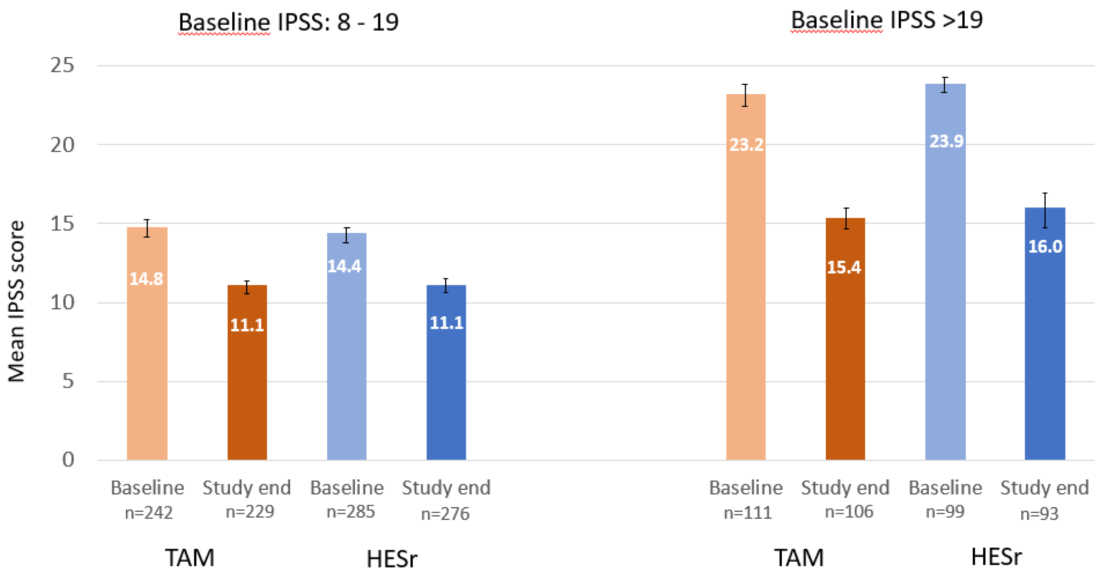

Figure S2. Mean (95% CI) IPSS total score at baseline and study end (6 months) for the treatment groups based on baseline symptom severity (iterative matching sample).
TAM: tamsulosin; HESr: hexanic extract of *Serenoa repens;* IPSS: International Prostate Symptom Score.

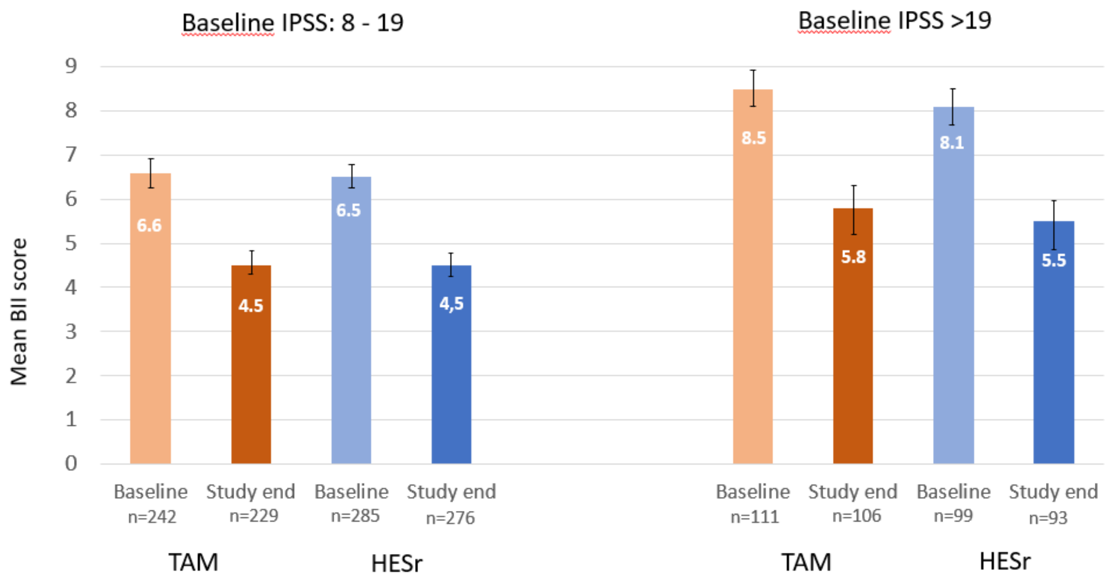

Figure S3. Mean (95% CI) BII score at baseline and study end (6 months) for the treatment groups based on baseline symptom severity (iterative matching sample).
TAM: tamsulosin; HESr: hexanic extract of *Serenoa repens;* IPSS: International Prostate Symptom Score; BII: Benign Prostatic Hyperplasia Impact Index.

Appendix

The following investigators participated in the QUALIPROST Study Group:

A Coruña: Dr. L. Busto Castañón; Dr. J.E. Duarte Novo; Dr. M. Montes Couceiro; Dr. A. Rodríguez Alonso; *Alicante:* Dr. J.A. Canovas Ivorra; Dr. C. López López; Dr. L. Prieto Chaparro; *Almería:* Dr. F. Gómez Berjón; Dr. J. Hortelano Parras; *Asturias:* Dr. A. M. Huescar; Dr. P.L. Muntañola Armora; *Badajoz:* Dr. E. Godoy Rubio; Dr. J. Mariño del Real; *Barcelona:* Dr. J. Armona Mani; Dr. F.J. Blasco Casares; Dr. S. Bucar Terrades; Dr. Ll. Cortadellas Angel; Dr. J. Fernández Zuazu; Dr. J.M. Malet Carreras; Dr. S. Mando Dakkar; Dr. J.M. Prats Puig; Dr. M. Puyol Pallás; Dr. J.A. Romero Martín; Dr. J. Sáenz de Cabezón Martí; Dr. J. Sánchez Macías; Dr. C. Vargas Blasco; Dr. J.M. Vayreda Martija; *Bilbao:* Dr. J.A. Gallego Sánchez; Dr. N. Prieto Ugidos; *Cádiz:* Dr. J.M. Arroyo Maestre; Dr. S. Garrido Insua; Dr. A. Gutiérrez de Pablo; Dr. M. Marmol Ruíz; Dr. F. Reyes Martínez; *Castellón:* Dr. J. Beltrán Persiva; Dr. L. Monzonis Rey; Dr. J. Palacios Castañeda; *Ciudad Real:* Dr. N. Jiménez López-Lucendo; Dr. D. Rodríguez Leal; *Córdoba:* Dr. J.C. Regueiro López; *Granada:* Dr. M. Cabezas Zamora; *Guipúzcoa:* Dr. G. Garmendia Olaizola; Dr. J.A. Rodríguez Andrés; *Jaén:* Dr. J. Jiménez Verdejo; Dr. E.J. Zarate Rodríguez; *León:* Dr. S.C. Gómez Cisneros; Dr. M. Lozano Rebollo; *Lleida:* Dr. J. Cortada Robert; Dr. L.M. Flavian Domenech; *Logroño:* Dr. A. Fernández Fernández; *Lugo:* Dr. F.J. Neira Pampin; *Madrid:* Dr. A. Abdallah Merhi; Dr. F. Arias Funes; Dr. I.T. Castillón Vela; Dr. J.M. Duarte Ojeda; Dr. M.J. García-Matres Cortés; Dr. J.F. Hermina Gutierrez; Dr. J.J. López-Tello García; Dr. C. Martín García; Dr. B. M.El-Awadeh; Dr. J.D. Rendón Sánchez; Dr. R. Rodríguez-Patron Rodríguez; Dr. J.C. Ruíz de la Roja; Dr. J. Vallejo Herrador; Dr. D. Vázquez Alba; *Málaga:* Dr. A. Bonilla Maldonado; Dr. J.M. Fernández Montero; Dr. A. Galacho Bech; Dr. C. Marchal Escalona; Dr. P. Rodero García; Dr. G. Sanz; Dr. J.D. Vázquez Cervilla; *Murcia:* Dr. G. Server Pastor; *Pontevedra:* Dr. D. Jamardo González; Dr. A. Selas Pérez; *Salamanca:* Dr. F. Díaz Alférez; Dr. M.F. Lorenzo Gómez; *Santander:* Dr. J.A. Portillo Martín; *Sevilla:* Dr. F.J. Giráldez Puig; Dr. M.A. Gutiérrez González; Dr. J.I. Huesa Martínez; Dr. Y. Ismail Tomaizeh; Dr. J. Leal Arenas; Dr. J. Martín Calero; Dr. J.L. Moyano Calvo; Dr. A. Ortiz Gámiz; Dr. J.M. Poyato Galán; Dr. J.A. Valero Puerta; Dr. E. Vilches Cocovi; *Tarragona:* Dr. P.L. Álvarez de la Red; Dr. J. Benagues Pamies; Dr. M. Prados Saavedra; *Tenerife:* Dr. T. Concepción Masip; Dr. C. Gómez de Segura Melcon; Dr. E. González de Chaves; Dr. J. Postius Robert; *Toledo:* Dr. F. Álvarez Fernández; Dr. A. Iglesias Justo; Dr. A. Melchor Galán; *Valencia:* Dr. J.E. Blasco Alfonso; Dr. M.A. Bonillo García; Dr. A. Collado Serra; Dr. J.A. García Cebrián; Dr. L.García Reboll; Dr. Y. Pallás Costa; Dr. M.J. Sánchez Sanchiz; Dr. J. Santamaría Meseguer; *Zaragoza:* Dr. A. García de Jalón Martínez; Dr. J.M. Gil Fabra; Dr. F. Monzón Alebesque; Dr. A. Ucar Terren.
